# Predictive processing as a scalable computational principle for embodied multitask intelligence

**DOI:** 10.1126/sciadv.aed7511

**Published:** 2026-08-14

**Authors:** Hayato Idei, Tamon Miyake, Tetsuya Ogata, Yuichi Yamashita

**Affiliations:** ^1^ Department of Information Medicine, National Institute of Neuroscience, National Center of Neurology and Psychiatry, Tokyo, Japan.; ^2^ Future Robotics Organization, Waseda University, Tokyo, Japan.; ^3^ Department of Intermedia Art and Science, Waseda University, Tokyo, Japan.

## Abstract

Humans exhibit remarkable flexibility in adapting to diverse and uncertain environments—a hallmark arising from the brain’s ability to integrate multimodal sensory streams into coherent predictive models. Drawing on this principle, we introduce a scalable hierarchical multimodal recurrent neural network grounded in predictive processing under the free-energy principle, capable of directly integrating more than 30,000-dimensional visuo-proprioceptive inputs without dimensionality reduction or handcrafted preprocessing. Using sensory data from teleoperation of a full-scale physical humanoid robot performing two caregiving-related tasks—rigid-body repositioning and flexible-towel wiping—the model learns to predict high-dimensional visuo-proprioceptive streams end to end. In open-loop adaptive inference experiments, the framework exhibits three emergent properties: (i) self-organized hierarchical latent dynamics governing task transitions, uncertainty, and occlusion inference; (ii) robustness to degraded vision via multimodal integration; and (iii) asymmetric interference in multitask learning. Although evaluated in simulations, the framework is extensible to closed-loop robot control, with proprioceptive predictions driving action, thereby establishing a generalizable computational foundation bridging brain theory, artificial intelligence, and embodied robotics.

## INTRODUCTION

Developing a unified computational framework that enables autonomous systems to flexibly learn and generalize across diverse sensorimotor behaviors is a central challenge in robotics and neuroscience. Such a framework is essential not only for realizing adaptive embodied intelligence but also for understanding the computational basis of human cognition and adaptability. One particularly urgent domain where this challenge manifests is long-term care. As societies worldwide age rapidly, the growing demand for caregiving is exacerbated by an increasingly severe shortage of professional caregivers (*1*–*3*). Physically demanding tasks such as patient repositioning or body cleaning are not only labor intensive but also a leading cause of musculoskeletal disorders, particularly lower-back pain, among caregivers (*4*, *5*). To address these challenges, a variety of assistive robotic technologies have been developed (*6*–*12*), including transfer devices, exoskeletons, and humanoid systems designed for lifting or repositioning patients. However, most existing systems are either intended to support human operators or are specialized for a single, narrowly defined task, limiting their applicability in the diverse and unpredictable situations of real care environments. Consequently, there is an urgent need for next-generation care robots capable of acting autonomously and flexibly across multiple tasks that involve close physical interaction with humans and substantial environmental uncertainty. Achieving such generalization remains a formidable challenge because caregiving behaviors—such as supporting body parts, adjusting posture, or wiping body surfaces—differ fundamentally in their motor dynamics, object interactions, and sensory feedback. A principled approach to generalizable sensorimotor learning is therefore required not only to advance care robotics but also to elucidate how adaptive intelligence emerges from multimodal sensory integration, with broader implications for real-world domains that demand flexible embodied cognition and control.

Humans can flexibly perform a wide variety of tasks with a single brain even under uncertain and dynamic conditions. Such cognitive flexibility is thought to arise from the brain’s remarkable capacity to efficiently process the continuous influx of massive, multimodal sensory signals and to contextualize them for adaptive behavior. An influential theoretical framework in neuroscience is predictive processing, a computational framework under the free-energy principle that explains cognition as the minimization of prediction errors between sensory inputs and internally generated predictions (*13*–*18*). Rather than passively receiving inputs, the brain continuously anticipates sensory signals and updates its internal models in response to prediction errors. In particular, applications of the free-energy principle to action and behavior are commonly referred to as active inference, in which behavior fulfills an agent’s predictions by changing its sensory inputs (e.g., altering joint states based on proprioceptive predictions) (*19*–*22*). Under this framework, prediction error minimization provides a unified account of perception (state estimation), action (control), and learning. This perspective has increasingly inspired research in artificial intelligence and robotics, where predictive processing–based neural architectures are being explored as a means of endowing robots with adaptive, brain-like learning capabilities across diverse tasks (*23*–*32*).

Despite this promise, a fundamental challenge remains: extending predictive processing to the direct integration of high-dimensional, multimodal sensory data, which is a prerequisite for robust multitask learning in real-world robotics. Current robotic approaches often rely on preprocessing, dimensionality reduction, or additional external processing modules, such as specialized transformations or attention-based mechanisms, to extract features from each modality (e.g., vision or proprioception), typically in a task-specific manner. Although effective in constrained or well-defined settings, such strategies inherently limit generalization because they depend on modality- and task-specific assumptions. This limitation becomes particularly problematic in multitask scenarios such as caregiving contexts, where tasks differ widely in motor patterns, sensory targets, and sources of uncertainty. These sources include interpatient variability, differences in physical dynamics (e.g., rigid versus deformable objects), environmental configurations (e.g., bed height and initial posture), and action timing. Collectively, these factors give rise to sources of uncertainty that are partially shared yet task specific. However, a unified computational framework that can directly integrate raw, high-dimensional, and uncertain sensory signals across modalities is lacking. Such a framework would not only enable robust and flexible learning across heterogeneous tasks but also validate predictive processing as a general computational principle for intelligent behavior under real-world uncertainty.

**Fig. 1.**
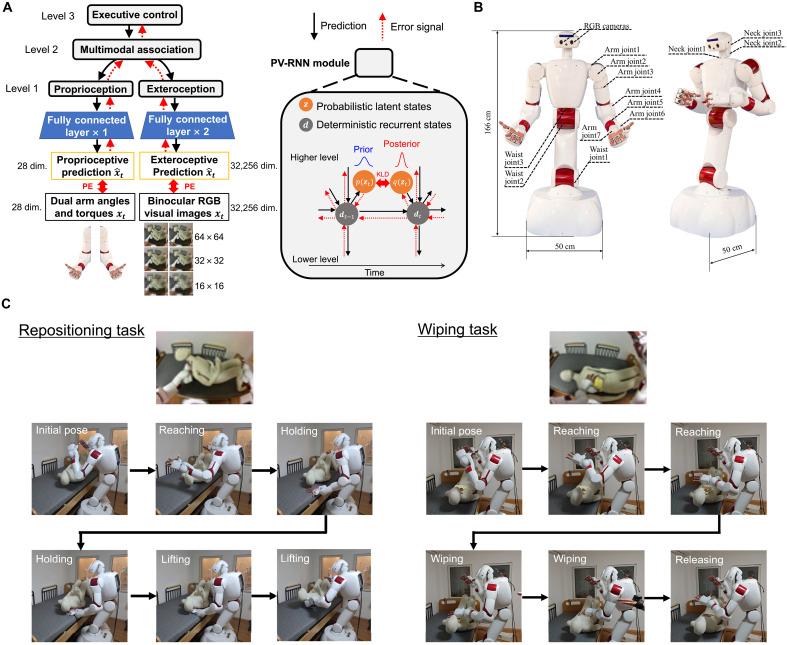
Computational framework and task setting. (**A**) Architecture of the scalable PV-RNN. During learning, sequential posteriors of latent states across all modules and time-invariant synaptic weights are updated by minimizing variational free-energy accumulated over all training sequences. PE: prediction error; KLD: Kullback-Leibler divergence. (**B**) The AIREC humanoid robot. (**C**) Two caregiving tasks used in this study: repositioning and wiping, both learned by the scalable PV-RNN using sensory data acquired via teleoperation of the physical AIREC humanoid robot and evaluated in simulations.

**Fig. 2.**
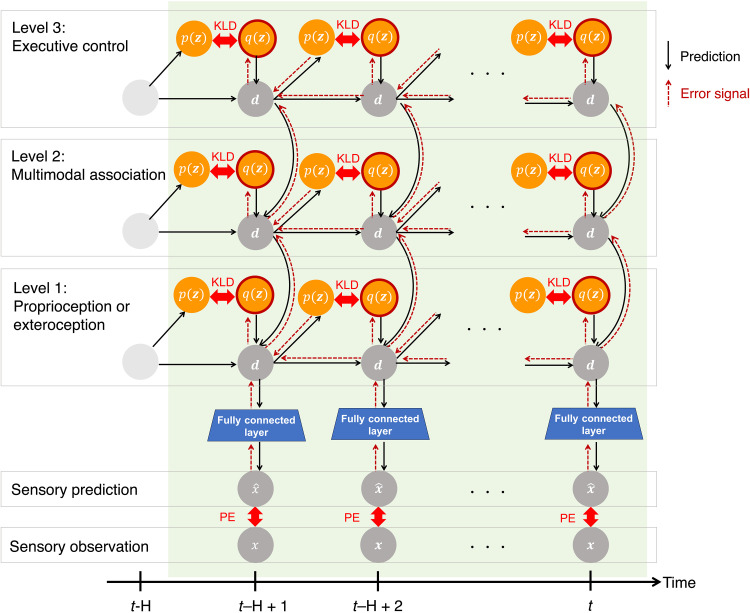
Temporal processing and online inference in the PV-RNN. During inference, posteriors in all modules are updated through minimization of variational free energy within a short time window (H), while synaptic weights remain fixed. For clarity, the distributed structure of the proprioceptive and exteroceptive modules is omitted.

Here, we introduce a scalable predictive coding–inspired variational recurrent neural network (PV-RNN) designed to integrate high-dimensional, multimodal sensory information. PV-RNN is a generative model that infers probabilistic and dynamic latent states under the free-energy principle, thereby capturing hidden stochastic structures in temporal data and remaining robust even in continuous and uncertain time series arising from human-robot interactions (*33*–*35*). In our earlier work, we extended PV-RNN into a hierarchical multimodal architecture and showed that it could self-organize dynamic modulations of hierarchical uncertainty estimation while accounting for complex neural phenomena such as sensory attenuation and allostasis, as studied in neuroscience (*36*, *37*). Building on this foundation, we refined the framework to enhance predictive performance/scalability by introducing additional feedforward processing, enabling the model to better handle high-dimensional sensory signals with rapid, jagged temporal variations, while still operating solely under the principle of prediction error minimization (Figs. 1A and 2). In contrast to conventional methods that depend on handcrafted features or dimensionality reduction, the proposed scalable PV-RNN learns end-to-end through variational free-energy minimization, which directly integrate raw, high-dimensional sensory signals across modalities. This capability enables the model to flexibly self-organize latent representations suited to diverse modalities and tasks, thereby offering a unified framework for multitask learning in robotics.

In summary, we implemented active inference and learning under a scalable and expressive generative model. The functional form of this generative model comprised a hierarchy of RNNs with conventional (tanh) activation functions. Crucially, a separation of temporal scales was introduced by successively decreasing the rate constants of the network dynamics at successively higher levels. Furthermore, lower levels were equipped with functional segregation by implicitly factorizing the generative model to predict different sensorimotor modalities (e.g., visual and proprioceptive). Note that, because of real-time computational constraints, we did not implement the scheme in a physical robot but focused on predicting visual and proprioceptive consequences of action. Specifically, we evaluated the model’s performance in open-loop simulations using visuo-proprioceptive data obtained from teleoperation of a full-scale physical humanoid robot performing caregiving-related tasks. In principle, the proprioceptive predictions could be used to drive synthetic motor reflexes in the spirit of reflexive active inference: i.e., in which proprioceptive predictions play the role of setpoints for closed-loop control of reflexes, in the spirit of the equilibrium point hypothesis (*38*, *39*). Beyond its engineering contributions, evaluating the scalable PV-RNN on caregiving tasks also provides scientific value, enabling us to test whether prediction error minimization, as a unitary computational principle, can support the dynamic processing of high-dimensional multimodal information and yield robust adaptive behavior under uncertainty, as observed in humans.

## RESULTS

### Task description

This study investigated robot learning in caregiving tasks through high-dimensional visuo-proprioceptive integration. We used the Dry-AIREC humanoid robot developed by Tokyo Robotics Inc., Tokyo, Japan (Fig. 1B). Dry-AIREC is equipped with multiple sensing modalities, including proprioceptive and visual sensors, providing a suitable platform for studying embodied interaction. The head was fitted with binocular cameras that captured red, green, and blue (RGB) images, and each arm had seven degrees of freedom with torque sensors at every joint. For input to the scalable PV-RNN, we used 28-dimensional proprioceptive signals (14 joint angles and torques) and 32,256-dimensional visual signals (binocular RGB images at 16-by-16, 32-by-32, and 64-by-64 resolutions).

The robot was positioned in front of a bed on which a mannequin (height: 1800 mm; shoulder width: 470 mm; leg length: 885 mm; arm length: 750 mm; and mass: 8 kg) served as the care recipient. We focused on two representative caregiving tasks, repositioning and wiping, that are central to maintaining the health and quality of life of individuals with limited mobility (Fig. 1C). The repositioning task (supine-to-sitting transfer) comprised three subtasks: (i) reaching the left hand to the mannequin’s neck (reaching), (ii) placing the left hand under the upper back while the right hand supported the legs (holding), and (iii) lifting the upper body (lifting). The wiping task also comprised three subtasks: (i) reaching for a towel placed on the mannequin (reaching), (ii) wiping the body surface (wiping), and (iii) completing the wiping motion (releasing).

The learning and test data were obtained through teleoperation using a motion-capture system under three bed heights (590, 650, and 710 mm). We recorded visuo-proprioceptive sequences for 18 learning trials (9 per task and 3 per height) and 6 test trials (3 per task and 1 per height). Figures S1 and S2 illustrate variability in visual signals across repetitions of the repositioning and wiping tasks. The robot’s sensory data were recorded at a sampling rate of 20 Hz, and the scalable PV-RNN generates one-step-ahead predictions at each time step (i.e., every 0.05 s) based on the observed sensory data.

The repositioning task required dynamic manipulation of a rigid but heavy body with shifting load distribution, while adapting to variations in bed height, initial posture, and motion timing. By contrast, the wiping task required manipulation of a flexible towel across curved and uneven surfaces, while also accommodating variations in bed height, towel position, initial posture, and motion pattern. Both tasks required accurate target reaching, adaptation to object properties, and robustness to diverse spatiotemporal uncertainties. Together, they represent qualitatively distinct sources of variability and rigid-body dynamics under load versus flexible-object surface interaction. The central question addressed in this study was whether a single computational model can simultaneously learn and generalize across heterogeneous tasks.

### Predictive processing of the neural network

The scalable PV-RNN is grounded in the free-energy principle; rather than directly consuming sensory inputs, it learns and infers by minimizing variational free energy across time steps (*36*)

$$\begin{array}{c} F_{t}=\underset{\text{Sensory prediction error}}{\underbrace{\frac{1}{2}{\left\|e_{t}\right\|}^{2}}}+W\sum_{l=1}^{3}\underset{\text{Influence of prior beliefs}}{\underbrace{D_{\mathrm{KL}}\left\{q\left[z_{t}^{\left(l\right)}|e_{t:T}\right]\left\|p[z_{t}^{\left(l\right)}|d_{t-1}^{\left(l\right)}]\right.\right\}}}\\ e_{t}=x_{t}-{\hat{x}}_{t} \end{array}$$ (1)

Here, $x_{t}$ denotes the observed sensory inputs, ${\hat{x}}_{t}$ the predicted sensory inputs, $e_{t}$ the prediction error in terms of the observed sensory inputs and the predicted sensory inputs, $d_{t}^{\left(l\right)}$ the states of recurrent units, $q\left[z_{t}^{\left(l\right)}|e_{t:T}\right]$ the posterior belief (latent states inferred from observations), and $p\left[z_{t}^{\left(l\right)}|d_{t-1}^{\left(l\right)}\right]$ the prior belief (latent states predicted from past experience) (time step t, last step T, and network level l). Variational free energy has two components, the first quantifies sensory prediction error, while the second is the Kullback-Leibler divergence (KLD) between posterior and prior, capturing how past experience constrains posterior inference. Statistically speaking, the prediction error term corresponds to accuracy while the influence of prior beliefs ensures a minimization of the complexity of the implicit generative model. The balancing hyperparameter W was set to 0.005, following our prior work on multimodal PV-RNNs (*36*). W controls the extent to which the posterior is updated in response to prediction errors, balancing the trade-off between adherence to prior beliefs and the reduction of prediction error.

During learning, both the time-invariant synaptic weights ω and the sequence-specific posterior beliefs $z_{0:T}^{\left(l\right)}$ are updated to minimize the cumulative free energy across entire learning sequences. Posterior beliefs are updated independently for each sequence, enabling the simultaneous learning of multiple tasks.

During testing, the synaptic weights remain fixed, and the model performs online inference by updating $z_{t-H+1:t}^{\left(l\right)}$ within a sliding short-time window (*H*) at each time step. This procedure locally minimizes the variational free energy, thereby implementing an adaptive inference process that dynamically adjusts the latent states to incoming sensory information.

Thus, the proposed scalable PV-RNN functions entirely through predictive processing based on variational free-energy minimization, without requiring handcrafted feature extraction, dimensionality reduction, or any additional external processing. For evaluation, we trained five scalable PV-RNNs with different random initializations to reconstruct visuo-proprioceptive sequences from 18 learning datasets. The trained networks were then tested in simulation-based generalization experiments using six test datasets, with five inference trials per sequence, yielding 30 test trials per trained network. All test evaluations were conducted in simulations rather than on a physical robot. In other words, we focused on predicting the proprioceptive and visual input streams under a variety of different conditions. However, we did not generate the visual or proprioceptive input online. Rather, we assumed that the proprioceptive predictions and visual consequences can be realized in a physical setting.

### Experiment 1: Multitask generalization through hierarchical probabilistic inference

**Fig. 3.**
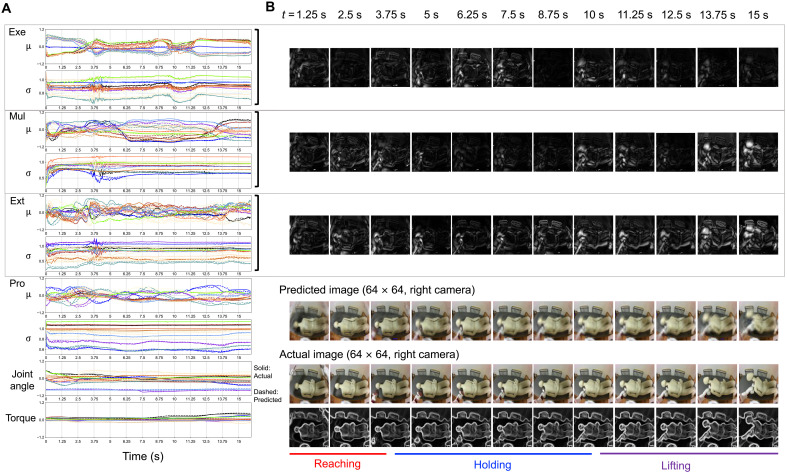
Hierarchical probabilistic inference during the repositioning task. (**A**) Dynamics of probabilistic latent states in each module, along with predicted and actual proprioceptive inputs. For the mean μ and sigma σ (SD) of latent states, posteriors and priors are shown as solid and dashed lines, respectively. For joint angles and torques, actual and predicted values are indicated by solid and dashed lines, respectively. Different colors represent distinct latent states, joint angles, or torques. For clarity of presentation, the time series of latent states are plotted for 15 selected states. Exe: Executive module. Mul: Multimodal associative module. Ext: Exteroceptive module. Pro: Proprioceptive module. (**B**) Visual representations in each module, together with predicted and actual visual inputs. For reference, an additional image emphasizing the contours of the actual visual inputs is shown beneath them. Contributions of latent states were analyzed through an ablation study. Brighter regions indicate greater influence of the corresponding module’s latent states on generating visual predictions. Results are shown for 64 by 64 images from the right camera.

Figure 3A illustrates an example of successful online inference for a test visuo-proprioceptive sequence in the repositioning task (movie S1). Figure 3B presents results of an ablation analysis quantifying how latent states in each network module contributed to visual predictions. Contribution strength is visualized in grayscale, with brighter (whiter) regions indicating image areas more strongly influenced by the ablated module (see Materials and Methods). Figure 3B enables comparison between the grayscale visualization of visual contributions from each module and the actual image, where contours are highlighted. These results revealed self-organized hierarchical task representations. The exteroceptive module maintained continuous visual representations, particularly highlighting regions corresponding to the robot’s left arm and the mannequin’s face. The multimodal associative module exhibited strong latent dynamics when the visuo-proprioceptive relationship was most prominent, for instance, as the robot’s hands approached the mannequin (0 to 3.75 s) or during the lifting phase (10 to 15 s). In contrast, the executive module showed marked changes in latent states and enhanced visual representations specifically at subtask boundaries, for example, at the onset of reaching (∼0 s), at the initiation of left- and right-hand movements during the holding phase (∼3.75 and ∼6.25 s), and at the beginning of the lifting phase (∼10 s). These patterns suggest that the executive module functioned as a controller for motion switching, selectively signaling transitions between distinct subtask segments.

**Fig. 4.**
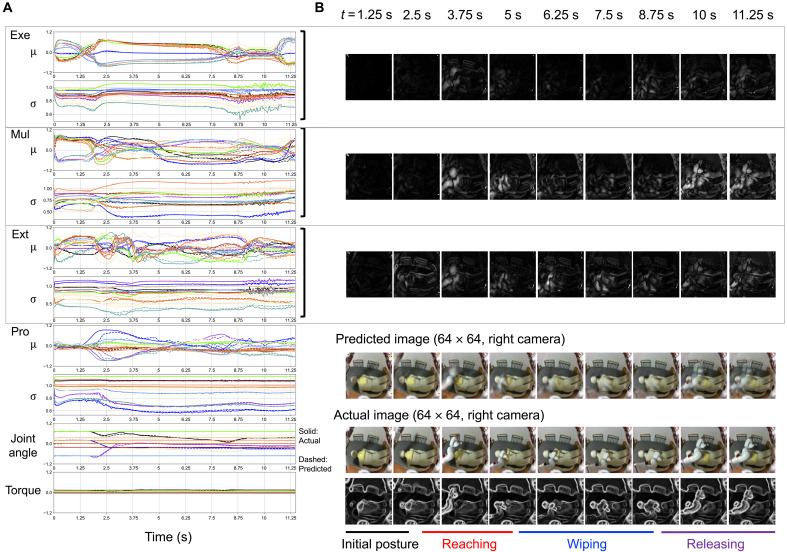
Hierarchical probabilistic inference during the wiping task. (**A**) Dynamics of probabilistic latent states in each module, along with predicted and actual proprioceptive inputs. For the mean μ and sigma σ (SD) of latent states, posteriors and priors are shown as solid and dashed lines, respectively. For joint angles and torques, actual and predicted values are indicated by solid and dashed lines, respectively. Different colors represent distinct latent states, joint angles, or torques. For clarity of presentation, the time series of latent states are plotted for 15 selected states. Exe: Executive module. Mul: Multimodal associative module. Ext: Exteroceptive module. Pro: Proprioceptive module. (**B**) Visual representations in each module, together with predicted and actual visual inputs. For reference, an additional image emphasizing the contours of the actual visual inputs is shown beneath them. Contributions of latent states were analyzed through an ablation study. Brighter regions indicate greater influence of the corresponding module’s latent states on generating visual predictions. Results are shown for 64 by 64 images from the right camera.

Figure 4 shows the corresponding results of the wiping task (movie S2). Overall, the three modules displayed similar hierarchical specializations as in the repositioning task. The exteroceptive module maintained continuous visual representations, particularly highlighting regions corresponding to the robot’s left arm and the mannequin’s body. The multimodal associative module captured dynamic visuo-proprioceptive integration, particularly during the reaching, wiping, and releasing phases (3.75 to 11.25 s). The executive module again exhibited state changes at subtask transitions, quickly detecting the shifts from initial posture to reaching and from wiping to releasing, while the transition from reaching to wiping appeared smoother. Notably, in predicted images (Fig. 4B), the mannequin’s body contours occluded by the robot arms were inferred, demonstrating the model’s capacity to infer hidden states in the absence of any direct sensory evidence. Collectively, these findings indicate that scalable PV-RNN evinced hierarchical inference, with each module assuming distinct roles in dynamically allocating task-dependent attention and controlling subtask transitions.

**Fig. 5.**
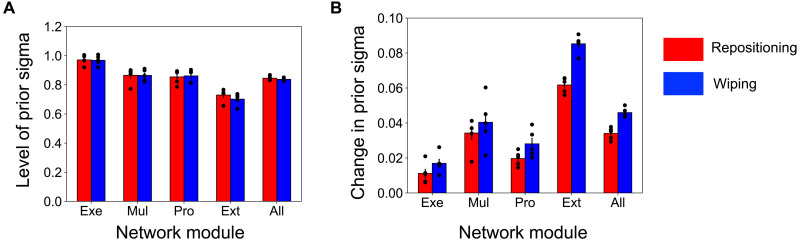
Estimated uncertainty and its temporal variability in repositioning and wiping tasks. (**A**) Mean levels of prior sigma estimated in each module. A paired *t* test indicated that the difference in mean prior sigma across all modules between the repositioning and wiping tasks was not significant [t(4)=1.67,P=0.17]. (**B**) Temporal changes in prior sigma, reflecting variability in uncertainty across time. A paired *t* test showed that the change in prior sigma averaged across all modules was significantly smaller in the repositioning task than in the wiping task [t(4)=−8.15,P=0.0012]. For both (A) and (B), values were first averaged across all time steps and latent states within each trial, and then averaged across five trials, three test sequences, and five independently trained networks for each task. Error bars represent SEs across the five networks. Exe: Executive module. Mul: Multimodal associative module. Ext: Exteroceptive module. Pro: Proprioceptive module. All: All modules. Error bars represent SEs across the five networks.

Last, Fig. 5 compares task-specific uncertainty dynamics estimated by the network. For the mean level of uncertainty (Fig. 5A), prior sigma values were first averaged across all time steps and latent states within each trial and then further averaged across 75 test runs for each task (five trials for each of the three test sequences and five independently trained networks). For variability in uncertainty (Fig. 5B), we quantified temporal fluctuations of prior sigma by computing, at each time step, the absolute difference from its value at the previous step, averaging these differences across all time steps and latent states within each trial, and then averaging them across the 75 test runs. Using these aggregated measures, we found that the mean level of estimated uncertainty was similar between repositioning and wiping, whereas variability in uncertainty was significantly greater for the wiping task. This suggests that the model flexibly adapted to differences in volatility across tasks, enabling generalization despite qualitatively distinct sources of uncertainty.

### Experiment 2: Robustness under uncertainty through multimodal integration

**Fig. 6.**
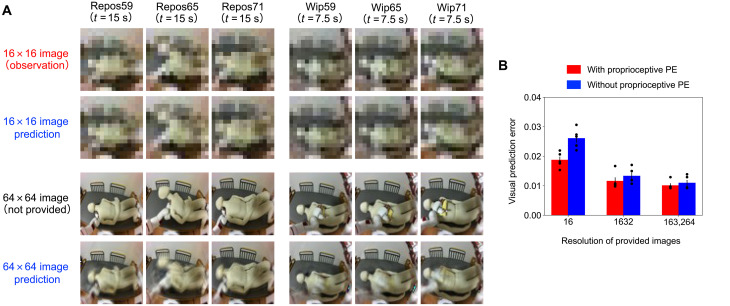
Robustness under limited visual inputs. (**A**) Examples of observed and predicted images at different resolutions. From top to bottom: observed 16 by 16 image, predicted 16 by 16 image, nonprovided 64 by 64 image, and predicted 64 by 64 image. Results are shown for three bed-height conditions (590, 650, and 710 mm) in both tasks. (**B**) Effects of proprioceptive input on visual prediction errors under different image-resolution conditions. Prediction errors were computed across all image resolutions and averaged over five trials, six test sequences, and five independently trained networks, separately for conditions with and without proprioceptive prediction error (PE). Error bars represent SEs across the five networks. A two-way nonparametric repeated-measures analysis of variance (ANOVA) using the aligned rank transform (ART-ANOVA) revealed significant main effects of image resolution [F(2,20)=105.57,P<0.001] and proprioceptive condition (with versus without proprioceptive PE) [F(1,20)=46.09,P<0.001], as well as a significant interaction between them [F(2,20)=13.57,P<0.001]. Post hoc pairwise comparisons with Holm correction confirmed that prediction errors were significantly lower in the presence of proprioceptive input than in its absence (P<0.001). A significant simple effect of proprioceptive condition was also observed when only 16 by 16 images were provided (P=0.034). Detailed Holm-corrected pairwise results for each image-resolution and proprioception condition are provided in table S1.

To evaluate whether our model maintains robustness under uncertain sensory conditions, we tested its performance when visual inputs were degraded in resolution. Figure 6A presents predicted 16-by-16 and 64-by-64 images when the model updated its latent states using only low-resolution (16-by-16) visual inputs in combination with proprioceptive inputs. For comparison across trials, results are aligned in time, revealing variability in motion timing and patterns among sequences. The model was able to generate high-resolution (64-by-64) visual predictions, although these signals were not provided during inference, accurately capturing task-dependent differences in timing and motion variability.

Figure 6B quantifies the effect of reduced visual resolution on the prediction error for full-resolution images. Values were first averaged across all time steps and sensory dimensions within each trial and then further averaged across 150 trials (five trials for each of six test sequences and five networks). In both the presence and absence of proprioceptive input, performance decreased with lower visual resolution; however, this decline was significantly mitigated when proprioceptive input was available (red versus blue bars in Fig. 6B).

These findings highlight the importance of multimodal predictive processing in ensuring robustness under both partial and uncertain sensory conditions. Once trained, the model can compensate for missing or degraded visual inputs by leveraging proprioceptive information, thereby sustaining accurate predictions. Moreover, the ability to operate with reduced-dimensional sensory inputs after training indicates the potential of our framework to achieve computational efficiency without sacrificing robustness.

### Experiment 3: Interference between tasks in learning

**Fig. 7.**
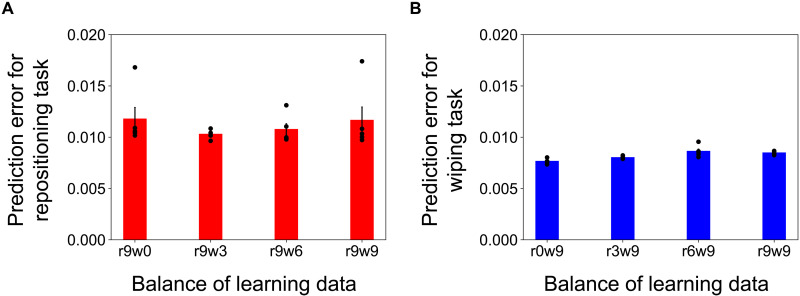
Interference between tasks in learning. (**A**) Prediction errors for the repositioning task under different data-balance conditions. The number of learning sequences for repositioning was fixed at nine, while the number of wiping sequences was varied. A one-way aligned rank transform ANOVA (ART-ANOVA) reported no significant differences in prediction error among data conditions [(3,16)=0.62,P=0.61]. (**B**) Prediction errors for the wiping task under different data-balance conditions. The number of learning sequences for wiping was fixed at nine, while the number of repositioning sequences was varied. A one-way ART-ANOVA indicated a significant effect of data condition on prediction error [F(3,16)=19.16,P<0.001]. Post hoc multiple comparisons using the Holm correction showed that the prediction error for the wiping task was significantly lower when the number of repositioning sequences was zero than when it was six (P<0.001) or nine (P<0.001). In addition, prediction error was significantly lower when the number of repositioning sequences was three than when it was six (P=0.0033) or nine (P=0.0033). For both (A) and (B), values are averaged across five trials, three test sequences, and five independently trained networks for each learning condition. Error bars indicate SEs across the five networks.

To investigate the influence of multitask learning on generalization, we examined the performance while varying the balance of learning data between the repositioning and wiping tasks. Figure 7A shows the prediction errors on test sequences of the repositioning task when the number of repositioning training sequences was fixed at nine, while the number of wiping training sequences was varied (0, 3, 6, and 9). Prediction error values were first averaged across all time steps and sensory dimensions, including both exteroceptive and proprioceptive domains within each trial and then further averaged across 75 trials (five trials for each of the three test sequences and five networks). Figure 7A demonstrates that increasing the amount of wiping data did not significantly influence the generalization performance in repositioning. This suggests that learning the wiping task did not interfere with the acquisition of task-specific representations required for repositioning.

In contrast, Fig. 7B shows that increasing the amount of repositioning training data led to a statistically significant rise in the mean prediction error for the wiping task; however, the effect was modest and not disruptive to overall generalization performance. These results indicate that the scalable PV-RNN maintained robust performance without a marked drop even when the number of learned tasks increased, while still suggesting an asymmetric pattern of task interference.

## DISCUSSION

This study introduced a brain-inspired framework for integrating high-dimensional, multimodal sensory information through a unified computational principle of prediction error minimization, implemented in a multimodal PV-RNN. By operating directly on raw sensory signals without task-specific preprocessing or external modules, the framework enabled flexible generalization across qualitatively distinct caregiving tasks. Using repositioning and wiping as representative cases, we demonstrated three key properties—(i) self-organization of hierarchical latent dynamics that regulated task-dependent allocation of attention, captured variability in uncertainty, and enabled the inference of occluded states; (ii) robustness under uncertain sensory conditions through visuo-proprioceptive integration; and (iii) asymmetric interference in multitask learning, where the more variable wiping task exerted minimal influence on repositioning, whereas learning the repositioning task resulted in a statistically significant but modest degradation in wiping performance, yet the model maintained overall robustness. Together, these results highlight the potential of predictive processing as a foundation for multitasking embodied intelligence.

In the present study, we conducted open-loop simulations of adaptive inference due to real-time computational constraints. Nevertheless, our framework is, in principle, extensible to closed-loop robotic control. In a closed-loop setting, the key difference is that predicted proprioceptive states (i.e., joint angles) are directly used to control the robot. Specifically, at each new sensorimotor time step, the initially predicted joint angles would serve as set points for a low-level controller (e.g., proportional-integral-derivative control), thereby driving the robot’s actual joint movements. The resulting proprioceptive feedback would then be incorporated as observations for subsequent online inference. In this manner, control and perception of joint angles occur concurrently, consistent with active inference schemes (*19*, *20*). Notably, similar closed-loop implementations have been demonstrated in prior work using PV-RNN (*35*, *36*). Although we did not perform full closed-loop task execution, which remains a limitation, the observed performance suggests that the approach is viable for control. We implemented an online inference scheme, demonstrating that the model can sequentially predict and reconstruct incoming visual inputs with only a limited number of posterior updates at each time step. In particular, we did not observe performance degradation under multitask learning conditions, and the model exhibited robustness under degraded visual inputs. These results indicate that the current level of predictive accuracy is already meaningful. While the feasibility of real-world deployment remains uncertain because of task-specific challenges such as handling heavy objects and requires further systematic evaluation, the present results suggest that the proposed framework has reached a stage where practical implementation can be explored.

Beyond the engineering importance, these findings provide a broader discussion on the computational principles of cognition (*40*–*43*). Predictive processing under the free-energy principle has been proposed as a unifying framework for perception and action. However, its capacity to process real-world, high-dimensional multimodal inputs has remained underexplored. Our results address this gap by demonstrating the computational validity of the principle in robotics, rather than strict biological fidelity. The model reproduced properties often observed in humans: hierarchical task representation (*44*–*49*), inference of occluded states, and reliance on proprioception to maintain robust object manipulation when visual information is ambiguous, such as in dimly lit environments. Moreover, the asymmetric interference between repositioning and wiping suggests that tasks with higher variability in uncertainty (such as wiping) may exert weaker influence on the learning of other tasks, whereas tasks with lower variability (such as repositioning) tend to have stronger effects. This asymmetry may be partly explained by differences in how variability in uncertainty shapes internal representations. Tasks with greater variability in uncertainty (such as wiping) may foster the formation of more flexible and generalized latent representations, enabling robust adaptation across contexts. In contrast, tasks with lower variability (such as repositioning) may lead to more specialized and precise representations, which can interfere with the learning of other tasks. This perspective provides insight into human developmental learning processes, suggesting that exploratory behaviors such as early motor babbling are effective for acquiring diverse skills without interfering with the learning of other behaviors (*50*–*52*). While speculative, these parallels suggest that our approach provides a promising computational tool to link neural principles with adaptive behavior in complex environments.

From an applied robotics perspective, the proposed framework offers practical advantages in three respects—robustness, as it can generalize across heterogeneous tasks without handcrafted preprocessing; efficiency, as it can later operate with reduced sensory resolution to lower computational costs; and scalability, as the same principle can be extended to additional tasks and modalities. These features underscore predictive processing as a versatile and principled pathway toward autonomous robots capable of addressing diverse real-world demands.

However, this study has several limitations. First, it was restricted to simulation-based evaluations using teleoperated data; real-time control experiments on physical robots were not conducted. Second, although effective, the online inference process is computationally demanding, running at approximately 3 Hz in our experimental computer environment when processing proprioceptive signals together with binocular 16 by 16 RGB inputs. Thus, it requires more efficient implementations for real-time operation. Third, the mannequin used as the care recipient was mechanically simplified (lighter and more stable than humans), reducing ecological validity. Addressing these limitations will require advances in real-time optimization, more realistic human-robot interaction scenarios, and scaling to broader caregiving and other contexts. As a future direction, incorporating physics simulators is a promising approach. We did not adopt physics-based simulation in the present study because of practical limitations in accurately reproducing the complex physical dynamics involved in our tasks. In particular, during the repositioning task, the robot exhibits variations in torque and joint states arising from subtle fluctuations in the mannequin’s posture and changes in physical contact, which are difficult to model faithfully in simulation. Similarly, in the wiping task, manipulating a flexible object (i.e., a towel) over a curved surface involves intricate deformable dynamics and contact variability that remain challenging to capture with current simulators. For these reasons, we rely on real-world teleoperated data in the present study. Nevertheless, integrating physics-based simulation with real-world data remains an important direction for future work, particularly for scaling data diversity and enabling closed-loop evaluation in more controlled settings.

Despite these constraints, our findings demonstrate that a brain-inspired computational framework can endow robots with robustness and versatility reminiscent of human cognition. Progress toward real-time implementations and scaling to richer task sets holds promise for the development of next-generation robots capable of supporting humans in diverse and uncertain environments. Such robots would not only alleviate the burden on people but also shed light on the computational principles that enable biological intelligence to thrive in complex worlds.

## MATERIALS AND METHODS

### Experimental design

The aim of this study was to develop and evaluate a brain-inspired framework for robust multitask learning. Specifically, we tested whether a single computational principle, prediction error minimization, can support the integration of high-dimensional, multimodal sensory information and enable generalization across qualitatively different tasks. To this end, we used a scalable PV-RNN, a hierarchical multimodal generative model designed to infer probabilistic latent states under the free-energy principle. Using this framework, we investigated whether a robot could simultaneously learn and generalize two distinct caregiving tasks, repositioning and wiping, that differ in their motor patterns, interaction objects, and sources of uncertainty. The present study did not involve real-time physical execution, but instead, all evaluations were conducted in simulation using visuo-proprioceptive data sequences recorded through teleoperation.

### Robot setup and data acquisition

Learning and test data were obtained by teleoperating a dual-arm humanoid robot with a motion-capture system. During task execution, proprioceptive signals (joint angles and torques from both arms) and exteroceptive signals (binocular RGB images) were recorded as time-series data. Each signal was normalized to the range −0.9 to 0.9 using its maximum and minimum values, consistent with the activation functions of the neural network. The two caregiving tasks were (i) repositioning, in which the robot reached toward and lifted the upper back of a mannequin, requiring the manipulation of a rigid body with varying load distribution; and (ii) wiping, in which the robot reached for and manipulated a towel to clean the mannequin’s curved surface, requiring control of a flexible object under variable contact conditions. Both tasks were performed under variability in initial mannequin posture, bed height, and action timing, thereby introducing spatiotemporal uncertainty into the visuo-proprioceptive data.

In total, 24 visuo-proprioceptive sequences were collected: 18 sequences (9 per task, 3 per bed height) for learning and 6 sequences (3 per task, 1 per bed height) for testing. To evaluate robustness across different initializations, we trained five scalable PV-RNNs with independently randomized synaptic weights. For testing, each trained model performed online inference across all test sequences with five independent trials, yielding 30 test trials per model.

### Prediction generation of the scalable PV-RNN

The scalable PV-RNN was organized into three hierarchical levels, consistent with our previous work. Level 1 encoded low-level exteroceptive and proprioceptive representations, level 2 integrated these modalities into mid-level sensorimotor representations, and level 3 controlled higher-level patterns of visuo-proprioceptive integration. To enhance the processing of high-dimensional sensory inputs, an additional feedforward neural network was inserted between level 1 and the sensory prediction outputs, enabling more flexible handling of complex signals.

Prediction generation was performed in a top-down manner through the network hierarchy. The internal state $h_{t,i}^{\left(s\right)}$ and output $d_{t,i}^{\left(s\right)}$ of the ith deterministic recurrent units of the sth target sequence at time step t is computed as

$$\begin{array}{c} h_{t,i}^{\left(s\right)}=\frac{1}{\tau }\left[\sum_{j\in I_{\text{Sd}}}\omega_{\mathrm{ij}}d_{t-1,j}^{\left(s\right)}+\sum_{j\in I_{\text{Sz}}}\omega_{\mathrm{ij}}z_{t,j}^{\left(s\right)}+\sum_{j\in I_{\text{Hd}}}\omega_{\mathrm{ij}}d_{t,j}^{\left(s\right)}+b_{i}\right]+(1-\frac{1}{\tau })h_{t-1,i}^{\left(s\right)}\\ \left(i\in I_{\text{Ed}},I_{\text{Pd}},I_{\text{Ad}},I_{\text{Cd}}\right) \end{array}$$ (2)

$$d_{t,i}^{\left(s\right)}=\text{tanh}\left[h_{t,i}^{\left(s\right)}\right]\;\left(i\in I_{\text{Ed}},I_{\text{Pd}},I_{\text{Ad}}\;I_{\text{Cd}}\right)$$ (3)

Here, $I_{\text{Ed}}$, $I_{\text{Pd}}$, $I_{\text{Ad}}$, and $I_{\text{Cd}}$ denote index sets of deterministic recurrent states in the exteroceptive, proprioceptive, multimodal associative, and executive modules, respectively. Similarly, $I_{\text{Ez}}$, $I_{\text{Pz}}$, $I_{\text{Az}}$, and $I_{\text{Cz}}$ denote index sets of probabilistic latent states. $I_{\text{Sd}}$, $I_{\text{Sz}}$, and $I_{\text{Hd}}$ denote recurrent states of the same module, latent states of the same module, and recurrent states of the higher-level module, respectively. $\omega_{\mathrm{ij}}$ is the synaptic weight from the jth to the ith neuron; $z_{t,j}^{\left(s\right)}$ is the output of the jth latent (posterior) state at time step t; τ is the time constant of the recurrent unit; and $b_{i}$ is the bias of the ith recurrent unit. A smaller τ yields faster-changing dynamics, while a larger τ produces slower dynamics. The initial internal states of the recurrent units $h_{0,i}^{\left(s\right)}$ ($i\in I_{\text{Ed}},I_{\text{Pd}},I_{\text{Ad}},I_{\text{Cd}}$) were set to 0 [thus, $d_{0,i}^{\left(s\right)}$ = 0].

The latent variable z follows a multivariate Gaussian distribution with diagonal covariance, implying independence between $z_{t,i}^{\left(s\right)}$ and $z_{t,j}^{\left(s\right)}$ for $i,j\in I_{\text{Ez}},I_{\text{Pz}},I_{\text{Az}},I_{\text{Cz}}\wedge i\neq j$. The prior distribution was parameterized from previous recurrent states in the same module

$$p\left[z_{t,i}^{\left(s\right)}\right]=p\left[z_{t,i}^{\left(s\right)}|d_{t-1,j}^{\left(s\right)}\right]=𝒩\left[z_{t,i}^{\left(s\right)};\mu_{t,i}^{\left(s\right)},\sigma_{t,i}^{\left(s\right)}\right]$$ (4)

$$\mu_{t,i}^{\left(s\right)}=\text{tanh}\left[\sum_{j}\omega_{\mathrm{ij}}d_{t-1,j}^{\left(s\right)}\right]$$ (5)

$$\sigma_{t,i}^{\left(s\right)}=\text{exp}\left[\sum_{j}\omega_{\mathrm{ij}}d_{t-1,j}^{\left(s\right)}\right]$$ (6)

where $\left(i\in I_{\text{Ez}}\wedge j\in I_{\text{Ed}}\right)\vee \left(i\in I_{\text{Pz}}\wedge j\in I_{\text{Pd}}\right)\vee \left(i\in I_{\text{Az}}\wedge j\in I_{\text{Ad}}\right)\vee \left(i\in I_{\text{Cz}}\wedge j\in I_{\text{Cd}}\right)$.

The posterior distribution was calculated as

$$q\left[z_{t,i}^{\left(s\right)}|e_{t:T^{\left(s\right)}}^{\left(s\right)}\right]=𝒩\left[z_{t,i}^{\left(s\right)};\mu_{t,i}^{\left(s\right)},\sigma_{t,i}^{\left(s\right)}\right]\left(i\in I_{\text{Ez}},I_{\text{Pz}},I_{\text{Az}},I_{\text{Cz}}\right)$$ (7)

$$\mu_{t,i}^{\left(s\right)}=\text{tanh}\left[a_{t,i,\mu }^{\left(s\right)}\right]$$ (8)

$$\sigma_{t,i}^{\left(s\right)}=\text{exp}\left[a_{t,i,\sigma }^{\left(s\right)}\right]$$ (9)

$$z_{t,i}^{\left(s\right)}=\mu_{t,i}^{\left(s\right)}+\sigma_{t,i}^{\left(s\right)}\times \epsilon ,\epsilon ∼𝒩\left(0,1\right)$$ (10)

Here, $a_{t}^{\left(s\right)}$ is the adaptive internal state of units parameterizing the posterior distributions. These adaptive variables $a_{t}^{\left(s\right)}$ were initialized from the corresponding prior distributions states before the training or inference process. ϵ is a random variable sampled from a standard normal distribution.

Two fully connected feedforward neural networks (FNN2L) for exteroceptive signals and one FNN1L for proprioceptive signals mapped recurrent states to predictions

$$f_{t,I_{\text{Ef}}}^{\left(s\right)}=\mathrm{FNN}2L\left[d_{t,I_{\text{Ed}}}^{\left(s\right)}\right]$$ (11)

$$f_{t,I_{\text{Pf}}}^{\left(s\right)}=\mathrm{FNN}1L\left[d_{t,I_{\text{Pd}}}^{\left(s\right)}\right]$$ (12)

$${\hat{x}}_{t,i}^{\left(s\right)}=\left\{\begin{array}{c} \text{tanh}\left[\sum_{j\in I_{\text{Ef}}}\omega_{\mathrm{ij}}f_{t,j}^{\left(s\right)}\right]\;\left(i\in I_{\text{Eo}}\right)\\ \text{tanh}\left[\sum_{j\in I_{\text{Pf}}}\omega_{\mathrm{ij}}f_{t,j}^{\left(s\right)}\right]\;\left(i\in I_{\text{Po}}\right) \end{array}\right.$$ (13)

Here, $I_{\text{Ef}}$ and $I_{\text{Pf}}$ denote index sets of units in the lower layer of the FNN2L and FNN1L, respectively. $I_{\text{Eo}}$ and $I_{\text{Po}}$ denote the output units for exteroceptive and proprioceptive predictions, respectively.

### Cost function of the scalable PV-RNN

Variational free-energy $F_{t}^{\left(s\right)}$ in the PV-RNN is formulated as

$$F_{t}^{\left(s\right)}=-\underset{\text{Accuracy}}{\underbrace{\text{log}p\left[x_{t}^{\left(s\right)}|d_{t}^{\left(s\right)}\right]}}+W\sum_{l=1}^{3}\underset{\text{Complexity}}{\underbrace{\left(D_{\mathrm{KL}}\left\{q\left[z_{t}^{\left(l\right),\left(s\right)}|e_{t:T}^{\left(s\right)}\right]\|p[z_{t}^{\left(l\right),\left(s\right)}|d_{t-1}^{\left(l\right),\left(s\right)}]\right\}\right)}}$$ (14)

The first term (negative accuracy term) corresponds to the negative log-likelihood. Assuming that each sensory state follows a Gaussian distribution with unit variance, this reduces to the squared prediction error between the observed sensations $x_{t}^{\left(s\right)}$ and predicted ${\hat{x}}_{t}^{\left(s\right)}$ sensations (constant term omitted)

$$-\text{Accuracy}=\sum_{i\in I_{\text{Eo}}\vee I_{\text{Po}}}\frac{1}{2}{\left[x_{t,i}^{\left(s\right)}-{\hat{x}}_{t,i}^{\left(s\right)}\right]}^{2}$$ (15)

In practice, the accuracy term was normalized by the dimensionality of each sensory modality.

The second term (complexity) is the KLD between posterior and prior latent distributions, quantifying the influence of prior beliefs. Assuming that both distributions are multivariate Gaussians with diagonal covariance, the KLD can be expressed analytically as

$$\text{Complexity}=\sum_{i}\left\{\text{log}\frac{\sigma_{t,i,p}^{\left(s\right)}}{\sigma_{t,i,q}^{\left(s\right)}}+\frac{{\left[\mu_{t,i,p}^{\left(s\right)}-\mu_{t,i,q}^{\left(s\right)}\right]}^{2}+{\left[\sigma_{t,i,q}^{\left(s\right)}\right]}^{2}}{2{\left[\sigma_{t,i,p}^{\left(s\right)}\right]}^{2}}-\frac{1}{2}\right\}$$ (16)

Here, $i\in I_{\text{Ez}},I_{\text{Pz}},I_{\text{Az}},I_{\text{Cz}}$. This complexity term was also normalized by the latent dimensionality of each module.

During learning, both synaptic weights ω and adaptive variables $a_{0:T^{\left(s\right)}}^{\left(s\right)}$ were updated to minimize cumulative free energy

$$F_{\text{learning}}=\sum_{s\in I_{s}}\sum_{t=1}^{T^{\left(s\right)}}F_{t}^{\left(s\right)}$$ (17)

This minimization used a (slow) gradient descent based on numerical derivatives. The free-energy gradients can always be expressed in terms of a prediction error. This is because the prior and posterior distributions (*p* and *q*) have a Gaussian form. In turn, this means the derivatives of their logarithms are always first order in the prediction errors.

During online inference, synaptic weights were fixed, and only adaptive variables were updated. Free energy was minimized within a sliding short-time window H

$$F_{\text{inference}}=\sum_{t^{\prime }=t-H+1}^{t}F_{t^{\prime }}$$ (18)

Adaptive variables within the time window were iteratively updated on the basis of this local free energy, with the window advancing at each time step. Crucially, this formulation enables the scalable PV-RNN to operate solely through variational free-energy minimization, without relying on handcrafted feature extraction, dimensionality reduction, or auxiliary processing modules external to the free-energy principle.

### Hyperparameter setting of the scalable PV-RNN

For simplicity, the dimensions of recurrent variables in the exteroceptive, proprioceptive, multimodal associative, and executive modules were set to $N_{\text{Ed}}=N_{\text{Pd}}=N_{\text{Ad}}=N_{\text{Cd}}=30$. Latent variable dimensions were set to $N_{\text{Ez}}=40$ and $N_{\text{Pz}}=N_{\text{Az}}=N_{\text{Cz}}=30$. These values were determined in a preliminary study that searched for the minimal configuration enabling successful reconstruction across all trained networks. Specifically, we first set identical dimensions for recurrent and latent variables (10, 20, or 30) and then increased the number of latent variables in the exteroceptive module to 40, which was necessary for adequate reconstruction. The fully connected layer in the proprioceptive pathway comprised 40 units, while the exteroceptive pathway included two fully connected layers with 40 and 50 units, respectively.

To incorporate a temporal hierarchy, we implemented multiple timescales across modules. In the lower perceptual modules, time constants were set as $\tau_{\text{Pd}}=\tau_{\text{Ed}}=2\;\text{or}\;4$ (half of the recurrent units each). In the multimodal associative module, $\tau_{\text{Ad}}=4\;\text{or}\;8$, and in the executive module $\tau_{\text{Cd}}=8\;\text{or}\;16$. Thus, higher-level modules exhibited slower neural dynamics than lower-level ones, realizing a hierarchical temporal structure.

Synaptic weights were initialized with the Xavier method (*53*). Biases of deterministic recurrent variables were initialized and fixed to random values drawn from a Gaussian distribution 𝒩(0,10), following a previous study (*54*). During learning, synaptic weights and adaptive (posterior) variables were updated for ${\mathrm{itr}}_{\text{learning}}=100,000$ iterations using the rectified Adam optimizer (*55*) with learning rate ${\mathrm{lr}}_{\text{learning}}=0.001$, $\beta_{1}=0.9$, $\beta_{2}=0.999$.

In online inference, only adaptive variables were updated. At each sensorimotor time step t, they were updated ${\mathrm{itr}}_{\text{inference}}=50$ times for a short time window of length H=30 time step (1.5 s) (or H=t if t<30), with learning rate ${\mathrm{lr}}_{\text{inference}}=1.0$. Parameter settings were selected by systematically searching combinations of ${\mathrm{lr}}_{\text{inference}}=\left\{1.0\right\}\times {\mathrm{itr}}_{\text{inference}}=\left\{20,30,40,50\right\}\times H=\left\{10,20,30,40,50\right\}$, choosing the minimal configuration that achieved successful reconstruction of the test data.

### Ablation study

To analyze how latent states at different hierarchical levels contributed to visual predictions, we conducted an ablation study during the online inference tests. For each test sequence, predicted images were generated under two conditions: (i) the standard condition, with all latent states intact, and (ii) the ablation condition, with latent states of a specific module removed. Predictions were averaged across 25 trials (5 test trials for each of the 5 independently trained networks). At each time step, the pixel-wise difference between the two conditions was computed. The averaged differences were visualized in grayscale, where brighter regions indicated visual areas most influenced by the ablated module. Figures 3B and 4B present the representative results from the right camera at 64-by-64 resolution, revealing distinct module-specific contributions to hierarchical task processing.

### Statistical analysis

Paired *t* tests were used to compare the mean and temporal changes in prior sigma (Fig. 5 and table S1). To analyze the effects of proprioceptive input on visual prediction errors under different image-resolution conditions, a two-way repeated-measures aligned rank transform analysis of variance (ART-ANOVA) with Holm-corrected post hoc tests was performed (Fig. 6 and tables S2 and S3). For examining prediction errors under different data-balance conditions, one-way ART-ANOVAs followed by Holm-corrected multiple comparisons were conducted (Fig. 7 and table S4). All statistical tests were two-tailed, and the significance level was set at P<0.05. Because this study involved an unprecedented computational simulation, it was difficult to estimate effect sizes in advance; therefore, no statistical methods were used to predetermine the sample size. Given the high reproducibility of the simulation, a minimum sample size of five networks was adopted. Even with this small sample, the analyses revealed clear statistical differences, suggesting that larger samples would not have substantially altered the main findings. Because of the limited sample size, these statistical tests were performed primarily as supportive quantitative analyses to complement the descriptive and comparative findings, rather than to draw definitive inferential conclusions. All data analyses were conducted using R software (version 4.5.1).
