# Supplementary material for: Predictive processing as a scalable computational principle for embodied multitask intelligence
Source: Sci Adv. 2026 Aug 14;12(33):eaed7511. doi: 10.1126/sciadv.aed7511 (PMC13475615; doi:10.1126/sciadv.aed7511)
Supplement: Supplementary file 1 [file sciadv.aed7511_sm.pdf]

Science Advances
AAAS

Supplementary Materials for

Predictive processing as a scalable computational principle for embodied multitask intelligence

Hayato Idei *et al.*

Corresponding author: Hayato Idei, idei@ncnp.go.jp; Yuichi Yamashita, yamay@ncnp.go.jp

Sci. Adv. **12**, eaed7511 (2026)
DOI: 10.1126/sciadv.aed7511

The PDF file includes:

Figs. S1 and S2
Legends for tables S1 to S4
Legends for movies S1 and S2

Other Supplementary Material for this manuscript includes the following:

Tables S1 to S4
Movies S1 and S2

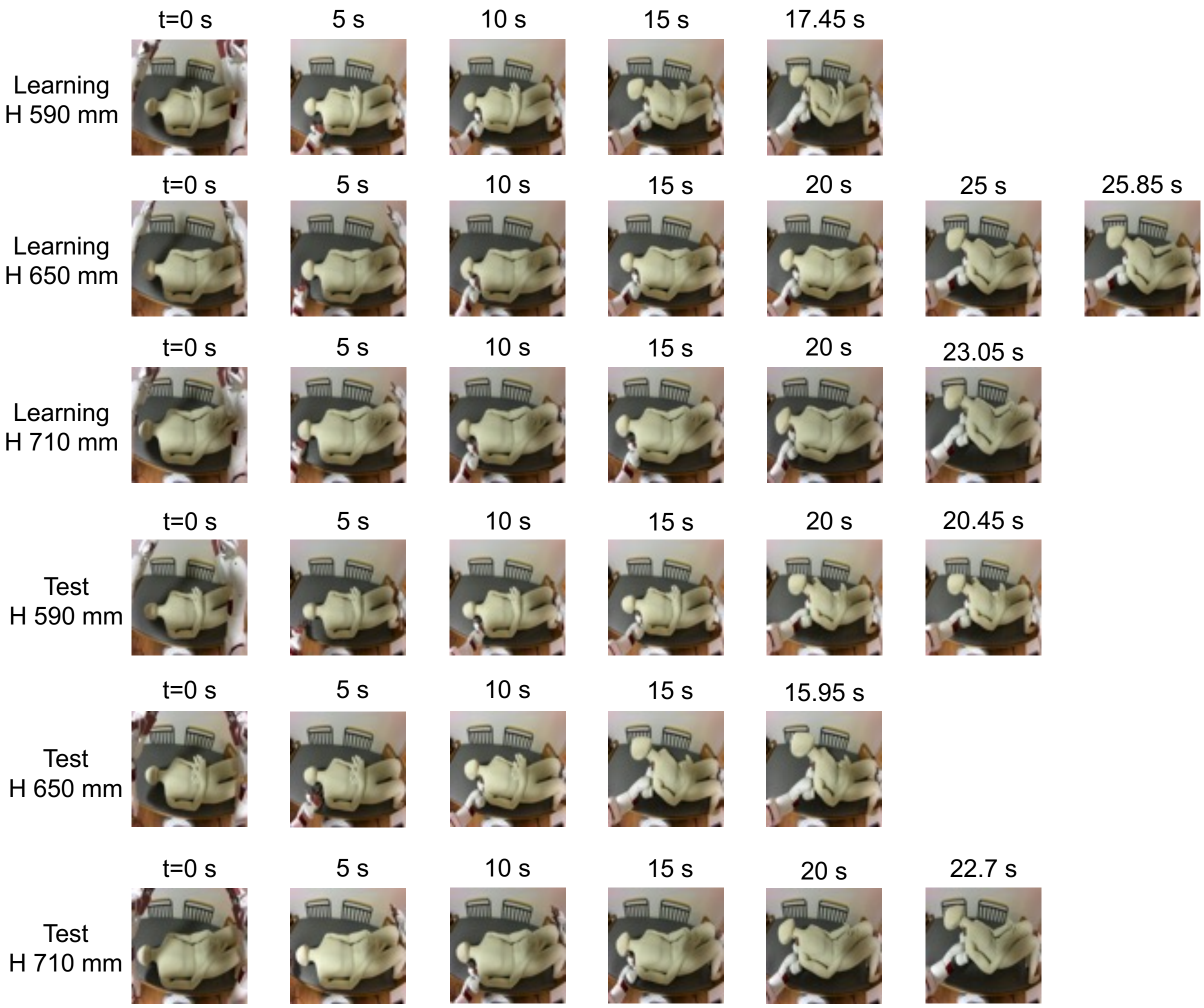

Fig. S1. Variability in visual signals across repetitions of the repositioning task.

The temporal evolution under three bed heights (590, 650, and 710 mm) is shown for both the learning and test datasets. For the learning data, three representative patterns out of nine are shown. The final image in each temporal sequence corresponds to the last time step. The visual inputs are shown for 64×64 images from the right camera.

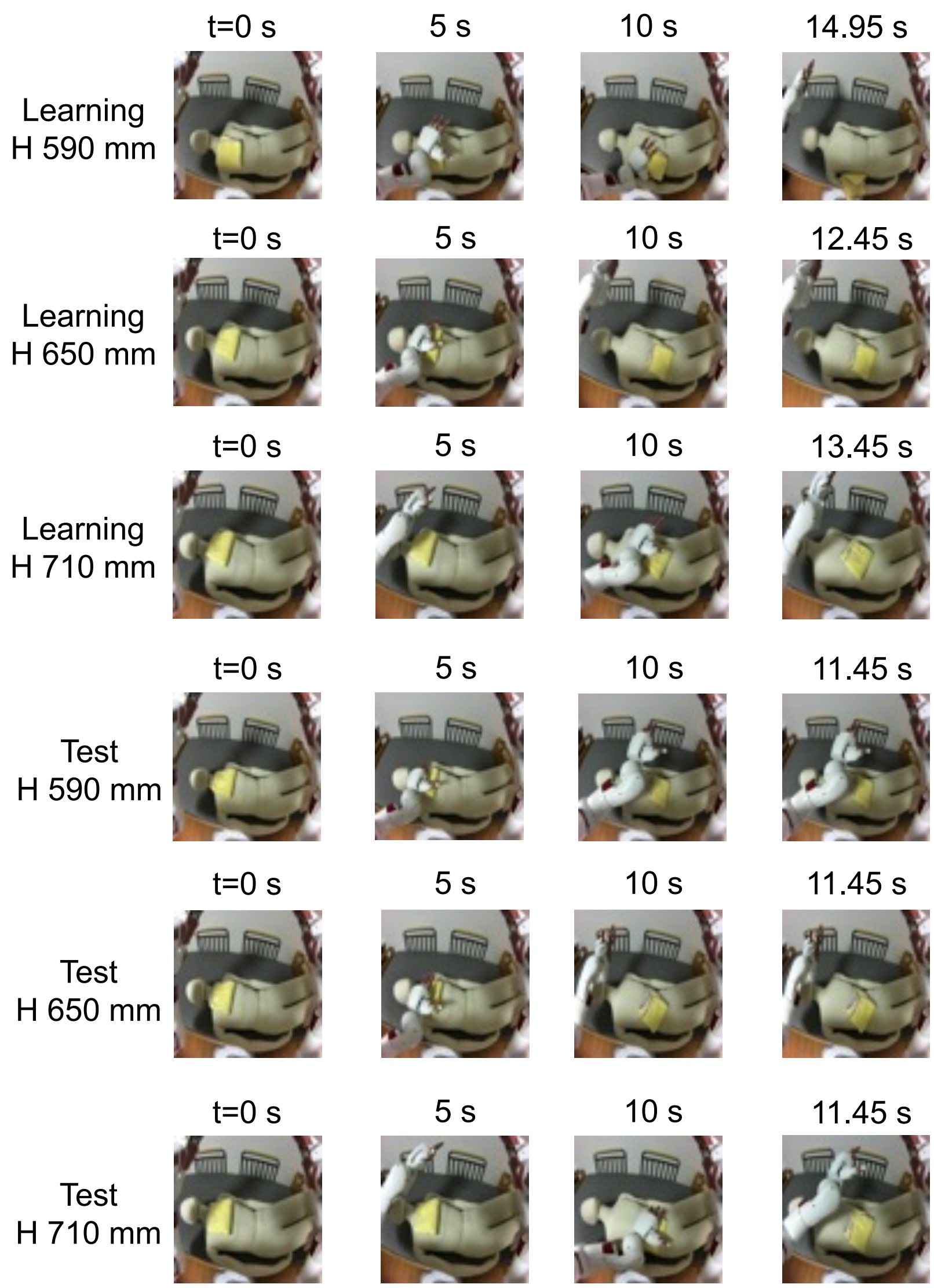

Fig. S2. Variability in visual signals across repetitions of the wiping task.

The temporal evolution under three bed heights (590, 650, and 710 mm) is shown for both the learning and test datasets. For the learning data, three representative patterns out of nine are shown. The final image in each temporal sequence corresponds to the last time step. The visual inputs are shown for 64×64 images from the right camera.

Table S1. Data values used for statistical tests of prior sigma level and prior sigma change.

Table S2. Data values used for statistical tests of visual prediction error under conditions with and without proprioceptive prediction error.

Table S3. Results of post-hoc pairwise comparisons for each condition defined by image resolution (low, middle, or high) and the presence or absence of proprioception (with or without).

Table S4. Data values used for statistical tests of prediction error under different data balance conditions

Movie S1. Visual predictions during the repositioning task.
Top: actual visual inputs at 64×64 resolution from the left (top-left) and right (top-right) cameras. Bottom: predicted visual images at 64×64 resolution from the left (bottom-left) and right (bottom-right) cameras generated by the scalable PV-RNN during online inference.

Movie S2. Visual predictions during the wiping task.
Top: actual visual inputs at 64×64 resolution from the left (top-left) and right (top-right) cameras. Bottom: predicted visual images at 64×64 resolution from the left (bottom-left) and right (bottom-right) cameras generated by the scalable PV-RNN during online inference.
